# The Nigerian Dietary Screening Tool: A Step toward Improved Patient-Clinician Communication in Nigerian Hospitals: A Pilot Implementation Study

**DOI:** 10.3390/nu16142286

**Published:** 2024-07-16

**Authors:** Nimisoere P. Batubo, Carolyn I. Auma, J. Bernadette Moore, Michael A. Zulyniak

**Affiliations:** 1 Nutritional Epidemiology Group, School of Food Science and Nutrition, University of Leeds, Leeds LS2 9JT, UK; fsnpb@leeds.ac.uk (N.P.B.); c.i.auma@leeds.ac.uk (C.I.A.); j.b.moore@leeds.ac.uk (J.B.M.); 2 Leeds Institute of Medical Research, Faculty of Medicine and Health, University of Leeds, Leeds LS2 9JT, UK; 3 Human Nutrition, University of British Columbia, Vancouver, BC V6T1Z4, Canada

**Keywords:** dietary assessment, Nigerian dietary screening tools, hypertension, implementation, patient–clinician communication, clinical practice

## Abstract

Implementing dietary screening tools into clinical practice has been challenging, including in Nigeria. This study evaluated the impact of the Nigerian dietary screening tool (NiDST) on patient–clinician communication and barriers to and facilitators of implementation. A mixed methods approach was used to collect data from patients (*n* = 151) and clinicians (*n* = 20) from outpatient clinics in Nigeria. Patients completed the validated 25-item NiDST prior to outpatient consultations. Both patients and clinicians completed the Measurement Instrument for Determinants of Innovations (MIDI) questionnaire to assess implementation determinants post-consultation. Semi-structured interviews were conducted for in-depth feedback. The fidelity of implementation was 92% for NiDST-reported dietary discussion, with a mean completion time of <6 min and an accepted marginal increase in consultation time (<10 min). For clinicians, 25% reported time constraints and their additional nutritional knowledge as barriers, while facilitators of NiDST implementation were the clarity and completeness of the NiDST, clinical relevance and improved patient–clinician communication, as reported by all the clinicians. Over 96% of patients reported the NiDST as quick to complete, with 90.7% reporting self-reflection on dietary intake. This study demonstrated the NiDST’s potential to enhance patient–clinician communication and highlighted major facilitators of implementation in clinical practice to improve dietary discussion in Nigeria.

## 1. Introduction

Hypertension is the leading risk factor for cardiovascular disease (CVD) and kidney disease worldwide, contributing to an estimated 11.3 million overall deaths and 10.8 million cardiovascular disease-related deaths in 2021 annually [1]. The burden of hypertension in low-and middle-income countries (LMIC), including sub-Saharan African countries, is rapidly increasing due to population growth and ageing, with a cumulative estimated prevalence of hypertension in Africa currently standing at approximately 30.8% [2,3,4]. In Nigeria alone, the most populated nation on the continent, hypertension has seen a threefold increase in prevalence from 1990 to 2019 when it passed 36% and affected more than one in three adults [5,6,7,8]. These estimates indicate that up to 28 million people in Nigeria are currently affected by hypertension [7]. Despite these figures, less than 11% of hypertensive adults in Nigeria achieve recommended blood pressure control as a result of poor access to healthcare, high cost of medication, non-adherence to pharmacological therapy, and poor diet (such as high intake of salt/sodium, unhealthy fats, refined sugars, and alcohol, coupled with low fruit and vegetable consumption) [7,9,10,11,12].

Diet plays a crucial role in health outcomes, particularly in the prevention and management of non-communicable diseases such as diabetes, obesity, hypertension, and cardiovascular disease [1,13]. In Nigeria, where the burden of these conditions is significant, the Nigerian government, alongside global health advisory groups (WHO and United Nations Assembly), reports that addressing dietary factors is paramount in the prevention of these non-communicable diseases (NCDs) [14]. The potential for substantial reductions in healthcare costs and improvements in quality of life through population-wide improvements in diet quality offers a compelling rationale for increasing the delivery of dietary assessment, nutrition education and dietary counselling by clinicians and healthcare professionals across various healthcare settings, including in Nigeria [15]. For instance, studies have demonstrated that higher diet quality among US adults is significantly and consistently associated with an 11–28% reduction in risk of all-cause mortality, including cardiovascular diseases (CVDs) and cancer, regardless of the educational and income level of the individual [16]. Similarly, a study conducted among 744 participants in the population-based cohort of the Moli-sani project demonstrated that higher nutritional knowledge was associated with higher adherence to the Mediterranean dietary pattern and a lowered prevalence of obesity among adults in the Southern Italian region [17]. Additionally, dietary screening tools have shown promise in measuring dietary changes associated with behavioural interventions, further supporting their integration into clinical practice [18]. This underscores the importance of integrating dietary assessment and counselling into clinical practice.

In this regard, validated dietary screening instruments, such as culturally appropriate Food Frequency Questionnaires (FFQs), offer standardised methods for comprehensively assessing dietary intake to inform evidence-based counselling [19,20,21]. In Nigeria, clinicians often lack culturally appropriate and effective strategies or tools to identify patients at risk and offer regionally specific dietary guidance to patients, and existing evidence-based recommendations may not be applicable to the diverse sociocultural landscape of Nigeria [22]. This gap underscores the necessity for culturally appropriate and validated dietary assessment tools that can be practically implemented in clinical practice for effective hypertension prevention and management in Nigeria. Despite their demonstrated utility, adopting these tools into frontline clinical practice has been challenging, including in Nigeria, necessitating robust tool development and validation and the implementation of research to facilitate integration. Several studies have highlighted that low participant satisfaction may hinder the adoption of validated dietary screening tools, which are crucial for providing personalised counselling and guiding management decisions in clinical care [23,24,25]. In addition, healthcare professionals recognise the potential benefits of these tools but report (i) a lack of effective region-specific dietary strategies for clinicians; (ii) inadequate time to evaluate dietary intake; (iii) an already heavy clinical workload; and (iv) low physician confidence in their nutrition knowledge [26,27,28,29,30,31]. However, studies report that brief dietary training, along with the use of a rapid/brief dietary assessment tool, can effectively diminish these barriers and enhance integration [19,32].

The concept of patient-centred care, which emphasises collaboration between patients and healthcare professionals in decision-making, has gained global recognition [19,33]. Effective patient–clinician interaction is central to this approach, allowing for effective information exchange, shared decision-making and mutual understanding of treatment goals. Globally, including in Nigeria, improved and clear communication between patients and clinicians is essential for assessing dietary habits, providing appropriate advice and promoting healthy behavioural change [34,35]. The Nigerian dietary screening tool (NiDST) is the first 25-item validated culturally-appropriate dietary screening tool developed for clinical use in Nigeria [21,31]. Our previous work demonstrates that the NiDST is a rapid (with a completion time of <8 min), effective and reliable tool for assessing and quantifying key food groups related to hypertension risk [21,31]. The NiDST represents a novel approach to enhancing patient–clinician communication regarding dietary habits in Nigerian hospitals. Unlike traditional methods that involve outpatient consultations without patient-reported outcome measures, NiDST offers a structured and culturally tailored framework for dietary assessment. This tool aims to address specific nutritional challenges faced by Nigerian patients and provide personalised dietary recommendations during clinical consultation. This pilot study aims to evaluate the practical clinical implementation of the NiDST specifically: (i) its perceived ability to enhance patient–clinician communication in nutritional education in real-world clinical settings; and (ii) the perceived barriers to and facilitators of its implementation into clinical practice in Nigeria.

## 2. Materials and Methods

### 2.1. Study Design and Setting

This study employed a single-centre, mixed-methods single-arm study with combined qualitative and quantitative approaches to gather data in two workstreams—(1) patients and (2) clinicians. Briefly, due to a lack of suitable dietary screening tools for use in the Nigerian clinical setting [31,36], clinicians do not routinely assess dietary intake in patients, so previous patient visits and clinical consults (without a standardised dietary screening tool) served as a retrospective control. This allowed patients and clinicians to reflect on their experience with the NiDST, compared to standard clinical practice (i.e., without the NiDST), to inform and foster patient–clinician discussions of dietary habits and recommendations to moderate hypertension risk. The study builds on methods and recruitment processes used successfully by the research team in earlier studies [31,36]. The study was conducted at the Rivers State University Teaching Hospital (RSUTH) in Port Harcourt, Nigeria. RSUTH serves as a tertiary healthcare facility, providing medical services to patients across Rivers State and neighbouring States, including Abia State, Bayelsa State, and Akwa Ibom State. The Family Medicine Department and Internal Medicine Department’s outpatient clinics were the primary sites for implementing the innovation and conducting data collection. Herewith, for ease of reporting, patient participants and clinician participants will be referred to as ‘patients’ and ‘clinicians’.

### 2.2. Sample Size

This study used a non-probability convenience sampling method to recruit participants (patients and clinicians) from the outpatient clinics at RSUTH in Nigeria. The sample size was determined based on Cohen’s guidelines for multiple regression analysis to detect a medium effect size (f2 = 0.15) with 80% power at a 5% significance level and five predictors using G*Power software (v 3.1.9.6) [37,38]. A minimum sample size of 92 patient participants was calculated. However, to account for a projected dropout rate of 20% and potential missing or incomplete data (as informed by our previous work [31]), we aimed for a target sample size of 150 participants [39,40]. In addition, 20 clinician participants were deemed adequate to capture the full range of qualitative questions and enable thematic saturation based on previous research suggesting that 9–30 participants are needed to reach saturation and informational redundancy in qualitative interview studies [41,42,43,44].

### 2.3. Participant Recruitment

The participants (patients and clinicians) were recruited over three weeks in December 2023. The patients were invited by recruitment posters within the hospital premises and through engaging with patients during their routine morning pre-consultation briefing sessions in the clinics. All interested patients were screened for eligibility using the inclusion and exclusion criteria used in our previous work [31] and outlined in Table 1. Before enrolment, each patient received a brief Participant Information Sheet and had the opportunity to communicate with the study team to ensure an understanding of the study protocol. Participants were also assured of (i) their voluntary participation rights; (ii) the option to withdraw from the study at any point without giving any reasons; and (iii) the confidentiality of their information. Clinicians were recruited based on their availability and involvement in providing medical care to patients with hypertension and cardiovascular diseases with ≥5 years of experience and were also assured of (i) their voluntary participation rights; (ii) the option to withdraw from the study at any point; and (iii) the confidentiality of their information. Written informed consent was obtained from all participants prior to study enrolment. To minimise bias, the hypertension state of participants was blinded.

### 2.4. Innovation—The Nigerian Dietary Screening Tool (NiDST)

**Figure 1:**
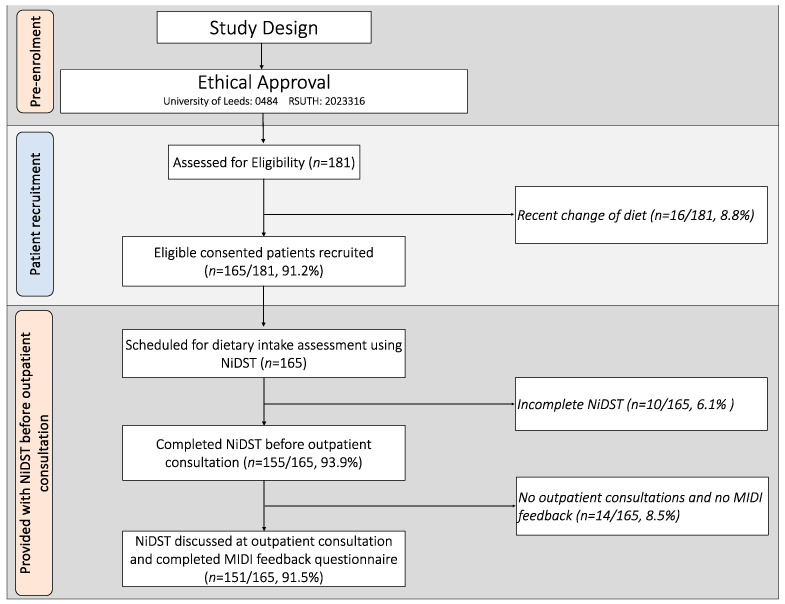
Flowchart of patients and fidelity of the NiDST during outpatient consultations. NiDST: Nigerian dietary screening tool; MIDI: Measurement Instrument for Determinants of Innovations.

The NiDST is a novel and validated dietary assessment method tailored for adults in Nigeria [21]. Briefly, the NiDST was validated by assessing the agreement between the average mean food group intake estimated by the NiDST and three repeat non-consecutive 24 h dietary recalls, with an average correlation coefficient of 0.60 among the food groups. In addition, the reproducibility of the NiDST was assessed by comparing the intake estimated by the first and second administration of the NiDST three weeks apart, with an average intra-class correlation coefficient of 0.77 among the food groups [21]. The NiDST is a validated, rapid and culturally appropriate dietary screening tool that collects patient dietary intake of 23 food groups (Table S1) over the past month through their selection of “rarely or never”, “1–2 times/week”, “3–5 times/week”, “daily”, “1–2 times/day”, “3–4 times/day”, or “5+ times/day” for each food group question. Implemented as a self-administered tool, patients can quickly complete the NiDST (mean < 8 min in validity testing) during the waiting period before their consultation [31]. Subsequently, clinicians can incorporate the patient-completed NiDST into the outpatient consultation for directing dietary discussions and offering personalised advice. This innovative but simple approach ensures a structured framework for dietary assessment and intervention, enhancing the effectiveness of hypertension management strategies (Figure 1).

### 2.5. Data Collection

We implemented the NiDST in a single-centre pilot study, enrolling patients who provided dietary information through the tool. Data collection took place over five weeks from December 2023 to January 2024 and was conducted in three (3) stages: pre-implementation (1 week), implementation (3 weeks), and post-implementation (1 week). This structure allowed us to assess the practical clinical implementation of the NiDST and gather comprehensive feedback from both patients and clinicians.

#### 2.5.1. Pre-Implementation Stage

During the pre-implementation stage, a structured questionnaire was used to collect baseline data from patients and clinicians. The patients’ questionnaire assessed sociodemographic information and medical history. Blood pressure, height, weight and waist circumference were also measured. Blood pressure was measured twice in the non-dominant arm using an automated mercury sphygmomanometer (model number: ZK-BB68, Shenzhen, China) following a standardised protocol with 5 min interval rests between measures. Height and weight were measured twice using a standard stadiometer (model number: DG2301, China) to ensure accuracy and waist circumference was measured twice with a measuring tape. Body mass index (BMI) was calculated from the values obtained for weight and height. The clinicians’ questionnaire focused on their experiences with dietary assessment and counselling prior to the implementation of NiDST. Additionally, to ensure proficiency in utilising the NiDST, all eligible consenting clinicians underwent a brief training session covering its administration, interpretation and integration into clinical practice.

#### 2.5.2. Implementation Stage

In the implementation stage, the NiDST was integrated into standard clinical workflow. Patients provided dietary information by completing the NiDST ahead of their outpatient consultation. Clinicians then reviewed the completed NiDST reports with each patient as part of their outpatient consultation to foster dietary discussion and deliver personalised dietary advice. This approach contrasts with the traditional methods that lacked structured patient-reported outcome measures. To gather feedback and assess experiences using the NiDST, a modified version of the Measurement Instrument for Determinants of Innovations (MIDI) was used (see Section 2.6.2) [45]. The MIDI was administrated to each patient after their outpatient consultation, while clinicians completed the MIDI at the end of the implementation stage. This provided insights into both patient and clinician perspectives on the NiDST’s utility and integration into clinical practice.

#### 2.5.3. Post-Implementations Stage

Subsequently, in the post-implementation stage, semi-structured interviews were conducted with a random subset of the clinicians (*n* = 15) and patients (*n* = 20) to garner an in-depth understanding of their experiences using the NiDST. Separate focus group discussions were then organised for clinicians (*n* = 10) and patients (*n* = 10) selected through a purposeful sampling approach. These discussions provided a platform for group interaction and exchange of shared experiences. These interviews and focus group sessions were scheduled to accommodate participants’ availability, with audio recordings made with their consent and later transcribed verbatim for analysis. This qualitative data collection aimed to identify perceived barriers and facilitators to the NiDST’s implementation, further informing future improvements and broader applications of the tool.

### 2.6. Outcome Measures

#### 2.6.1. Fidelity of Implementation

The fidelity of the use of the NiDST for the dietary assessment of individuals was determined by the percentage of (i) patients recruited for the study; (ii) patients who completed the NiDST before an outpatient consultation; and (iii) NiDST discussed at outpatient consultation and MIDI feedback provided.

#### 2.6.2. Barriers to and Facilitators of Implementation

The Measurement Instrument for Determinants of Innovations (MIDI) supports the designing of new implementation strategies within clinical care settings [45,46]. A modified version of the MIDI (Table S2) was completed by both clinicians and patients and serves to garner our understanding of critical determinants that may affect the implementation of NiDST use in clinical settings [45]. The modified MIDI comprises four scales, measuring determinants for implementations related to (i) the innovation—the NiDST (five determinants); and (ii) potential user of the innovation [i.e., clinicians (eight determinants); and (iii) patients (seven determinants)] with answer categories ranging from “totally disagree”, “disagree”, “neutral”, “agree” and “totally agree”. Notably, we did not evaluate the “organisational determinants” and “sociopolitical determinants” due to (i) the clinicians representing the organisation as their place of work; and (ii) the study’s main focus of assessing clinician–patient-level barriers and facilitators to implementation.

### 2.7. Data Analyses

#### 2.7.1. Quantitative Data

The study data from the clinicians and patients were anonymised and entered into Microsoft Excel for quality assurance purposes prior to analysis. Patient data were stratified into hypertensive and non-hypertensive groups. Descriptive statistics, including frequencies, percentages, means and standard deviations, were used to summarise the details of the sociodemographic, fidelity, medical history, clinical characteristics such as blood pressure, body mass index (BMI), height, weight, waist circumference, NiDST completion time, and consultation time. Chi-square tests (for categorical variables) and independent-sample t-tests (for quantitative variables) were performed to compare variables between hypertension and non-hypertension groups. MIDI determinants answered by ≥20% of clinicians and patients with “totally disagree/disagree” were considered barriers, while those answered by ≥80% with “agree/totally agree” were considered facilitators. Quantitative data were analysed using an R computing environment (version 4.3.1) [47].

#### 2.7.2. Qualitative Data

Audio recordings of interviews and focus group discussions were transcribed, checked, and anonymised and were imported into NVivo 14. They were coded by the first author (NPB) using the interview question domains (primary codes) and then for emergent themes (secondary codes) through repeated analysis of the content [48]. The reliability of coding was evaluated by a second coder (not involved in the data collection), resulting in an initial agreement of 98%, with resolution of discrepant code through discussion. Once the final coding framework was established, both coders independently indexed transcript segments to appropriate themes and subthemes using NVivo software, version 14 [49]. The qualitative data were then summarised and described narratively, utilising illustrative anonymous quotations to encapsulate participants’ experiences, perspectives and suggestions regarding the use of the NiDST. Qualitative data analysis was performed using NVivo software (version 14) [49].

## 3. Results

### 3.1. Participant Characteristics

A total of 181 patients were screened for eligibility. In total, 16 were excluded due to a recent change in diet, while 165 satisfied the inclusion criteria and were enrolled to participate in the study after providing informed consent (enrolment rate of 91%) (Figure 1). The mean age of the patients was 44.4 ± 10.2 years. Hypertensive and non-hypertensive individuals did not differ by sociodemographic characteristics, body mass index, waist circumference or physical activity levels (*p* > 0.05) (Table 2). However, there was a significantly higher proportion of men among the hypertensive group (59.1%) compared to the non-hypertensive group (36.5%) (*p* = 0.010) (Table 2). As expected, hypertensive patients had significantly higher mean systolic, diastolic blood pressure and mean arterial pressure (*p* < 0.001) compared to non-hypertensive patients (Table 2). Twenty (20) clinicians who provided outpatient consultation had an average age of 42.6 ± 6.0 years and an average of 13.0 ± 4.7 years of experience. The specialities represented were family medicine (55%) and internal medicine (45%).

### 3.2. Food Intake Assessment

The mean daily intakes (servings/day) of the 23 food groups assessed using the NiDST are shown in Table 3. Notably, there were no significant differences (*p* > 0.05) in the intake of the 12 food groups commonly considered “healthy” between hypertensive and non-hypertensive participants. However, hypertensive individuals reported significantly higher (*p* < 0.05) intakes of 11 food groups commonly considered “unhealthy”, compared to non-hypertensive participants. These unhealthy food groups included red meat, eggs, processed meat, fried and fast foods, soups and stews, desserts and sweets, soft drinks, alcoholic drinks, and salt/seasonings (Table 3).

### 3.3. Completion and Consultation Time

The mean NiDST completion time was 5.5 ± 1.7 min, and no difference was observed between hypertensive (5.5 ± 1.6 min) and non-hypertensive groups (5.5 ± 1.7 min) (*p* > 0.05) (Table 2). Additionally, utilisation of the tool during outpatient consultation for dietary counselling increased overall consultation time by an average of 9.6 ± 1.5 min with no significant difference between hypertensive (9.6 ± 1.5 min) and non-hypertensive groups (9.6 ± 1.5 min) (*p* > 0.05) (Table 2). This short completion time and additional consultation time represent a manageable increase in outpatient consultation (i.e., 15–20 min), which can be further streamlined to reduce time required and insure a seamless integration of the NiDST into the clinical workflow.

### 3.4. Fidelity of the Use of the NiDST

As shown in Figure 1, the fidelity of patient recruitment for the implementation of the NiDST was 91.2%, out of which 93.9% completed the NiDST and were scheduled for outpatient consultation and dietary advice. All the completed NiDSTs were discussed during each patient outpatient consultation. In addition, 91.5% of the patients completed the study protocols (i.e., completion of NiDST, outpatient consultations, and the MIDI feedback questionnaire). These findings indicate that the recruitment, screening, and consultation phases of the study were successfully implemented with high adherence.

### 3.5. Barriers to and Facilitators of Implementation of the NiDST

**Figure 2:**
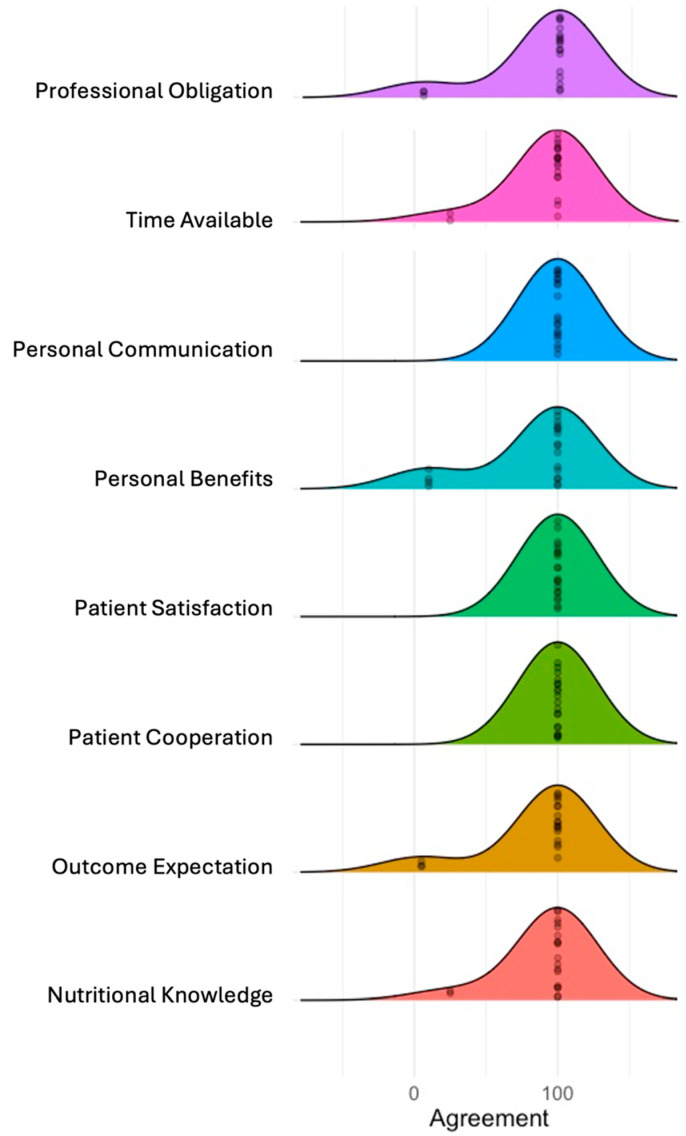
Clinicians reported determinants of implementation using MIDI determinants. The ridgeline plot displays the distribution of agreement levels for the MIDI determinants. Each coloured ridge represents the distribution of responses for a determinant, with the width of each curve indicating the spread of agreement levels, and the peak represents the most frequent level of agreement among respondents.

The clinician-reported factors related to the use of the NiDST to assess patient dietary intake and inform dietary advice in routine clinic visits are presented in Table 4 and Figure 2.

#### 3.5.1. Barriers Perceived by Clinicians

Notably, no barriers to the implementation of NiDST were reported by patients. The most common barriers reported by clinicians were (i) inadequate nutritional knowledge (25% indicated not having enough knowledge to use and interpret the dietary assessment tool); and (ii) time constraints (25% indicated not having enough time to assess patient dietary intake and offer dietary counselling to patients) (Table 4 and Figure 2). A frequently expressed concern was the fact that an additional time of ~10 min (Table 2) is needed to offer personalised dietary advice to patients, which may not be feasible in busy clinics due to time constraints, as illustrated by one clinician:

*“We only offer little dietary support to patients in the clinic. The number of patients we see on each clinic day is so much as such there is no time to fully assess patient food intake and provide one-on-one dietary counselling. Personally, I refer patients to see the dietician for the purpose of nutritional assessment and counselling”. (Female, 28 years).*

Another clinician stated that:

*“In my opinion, the tool was very useful, but the challenge is the additional time needed to counsel and make recommendations to the patients because you can imagine spending 20–45 min with one patient in a busy clinic”. (Male, 38 years)*

Similar sentiments were expressed by another clinician, who remarked that:

*“Well, it takes me some time to go through the tool and understand the patient’s food intake before I can start discussing it with them and advise them, but I think if the tool can be made simple and training be given on how to use it, then it will be easy to apply it without taken much time”. (Male, 36 years)*

#### 3.5.2. Facilitators Perceived by Clinicians

Encouragingly, despite some perceived barriers reported by some clinicians, an overwhelming majority (85%) of clinicians agreed that the NiDST is compatible with their clinic routines (Table 4). The procedural clarity, completeness of the food list and the simplicity of questions were mentioned as facilitating attributes of the NiDST, with all the clinicians reporting a high level of clarity and understanding of the NiDST, as illustrated by one clinician’s narrative:

*“For me, the tool integrated well into our routine workflow and training clinicians on how to use the tool, especially how to interpret the tool provided some sort of dietary guidelines for each food group. Yeah, this can help clinicians more focused on the dietary discussion with the patient, and this can shorten the consultation time” (Female, 48 years)*

Furthermore, the clinician-reported facilitators of the utilisation of the NiDST were (i) personal benefits—where 90% reported that the NiDST supported them in assessing patient dietary intake and provided better engagement and communication with patients); (ii) outcome expectations—95% indicated that the NiDST supported them to offer dietary advice to patients); (iii) professional obligation—95% expressed dietary intake assessment and providing dietary advice to patients as their routine task); and (iv) patient satisfaction—all clinicians indicated that patients were satisfied when using the tool with their dietary intake (Table 4 and Figure 2). Overall, these findings suggest that the NiDST can support clinicians in understanding patients’ dietary intake and offer personalised dietary advice. In this regard, one of the clinicians remarked:

*“From my experience, I’ve found that the NiDST really helps me dive into understanding my patients’ dietary habits better. It’s like having a comprehensive roadmap right in front of me during consultations. And you know what’s great? It doesn’t just stop there. Using the NiDST has actually improved my communication with patients. It’s like it bridges that gap between us, making our conversations more meaningful and productive. It’s been a game-changer for me, honestly”. (Female, 42 years)*

Likewise, another clinician described:

*“Yeah, I saw most of the patient were satisfied because the dietary discussion was focused on their usual food intake. They were able to ask questions on the right food they eat to reduce their risk of hypertension. Like, there was this one patient who mentioned how surprised to know that excess use of Maggi can worsen hypertension. They were like, ‘I use plenty Maggi when cooking my stews and soups”. (Female, 36 years)*

#### 3.5.3. Facilitators Perceived by Patients

**Figure 3:**
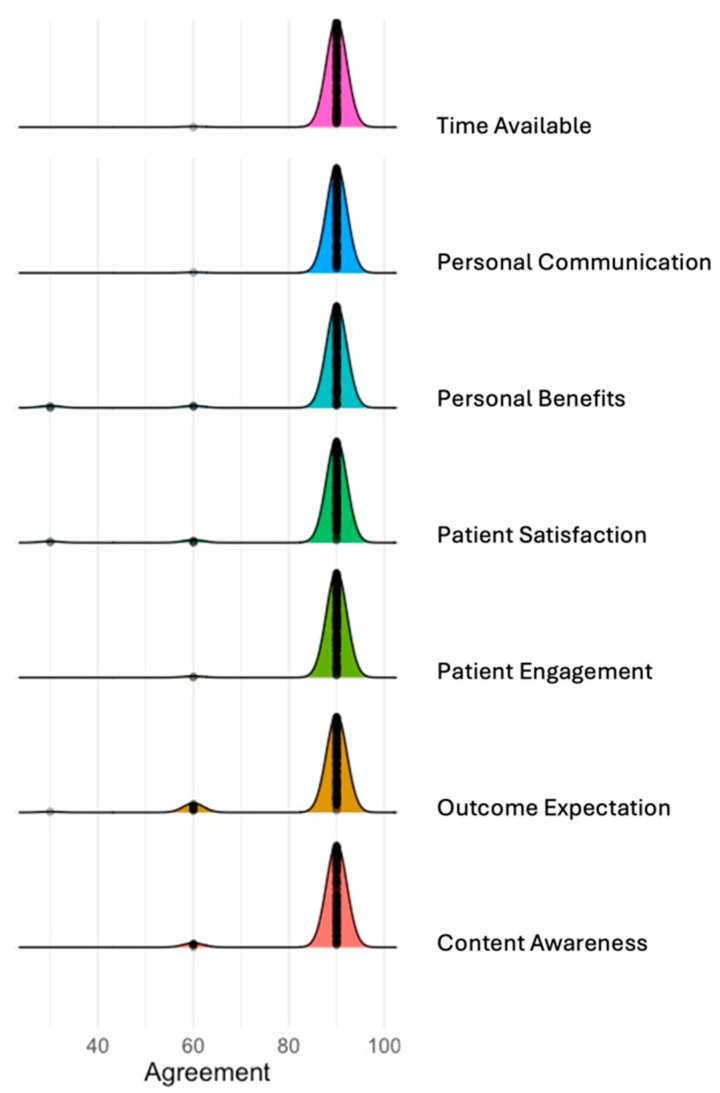
Patients reported determinants of implementation using MIDI determinants. The ridgeline plot displays the distribution of agreement levels for the MIDI determinants. Each coloured ridge represents the distribution of responses for a determinant, with the width of each curve indicating the spread of agreement levels, and the peak represents the most frequent level of agreement among respondents.

Table 5 and Figure 3 present the patient-reported facilitators related to using the NiDST to assess their dietary intake and support clinicians in providing dietary advice during routine clinic visits. Firstly, a significant proportion of the patients (≥96%) were satisfied with the format, content and time used to complete the NiDST (Table 5 and Figure 3), with a completion time of ~6 min, as illustrated in Table 2. For example, one female hypertensive patient described the ease with which she completed the assessment:

*“[It] was very straightforward and easy to fill out. The instructions at the top clearly explained what was needed, and the arrangement with the columns was simple to understand”. (Female, 45 years, >5 years hypertensive).*

In the same way, an older male patient stated:

*“I was able to complete the questionnaire in just a few minutes while waiting to see my doctor. It didn’t feel long or burdensome at all. The length and time needed fit perfectly into the normal clinic visit”. (Male, 61 years, non-hypertensive)*

Secondly, the majority of the patients (≥96%) found the NiDST to be beneficial to them as it (i) was supportive for clinicians to have an in-depth understanding of their dietary intake (98.7%); (ii) improved their engagement and communication with clinicians on dietary discussion (≥98%); and (iii) increased self-awareness of their food intake (91%) (Table 4). Lastly, 96% of patients expressed satisfaction with both using the screening tool and the consultation process (Table 5 and Figure 3). On this note, a patient stated that:

*“Filling the form and discussing with my doctor made me more aware of how often I eat food prepared by mama put and red meats each week. It was eye-opening, and this will motivate me to improve my diet. For me, I will complete the form next time I come to the hospital” (Male, 47 years, 1 year hypertensive)*

Another hypertensive patient indicated having an improved dietary counselling session as a result:

*“I think the use of the complete form really helps my doctor to understand the food I usually eat. I was really happy to hear the doctor telling me to reduce some food that I eat because I can contribute to my BP not being controlled”. (Female, 56 years > 5 years hypertensive)*

## 4. Discussion

In this present study, we implemented a culturally appropriate Nigerian dietary screening tool (NiDST) into outpatient clinic workflow in a pilot study to assess dietary intake in adults with or without hypertension to evaluate (i) its effectiveness in enhancing patient–clinician interaction and dietary counselling; (ii) the perceived benefit of the tool; and (iii) clinicians’ and patients’ perceived barriers to and facilitators of implementation in a Nigerian hospital. Our study, conducted in a Nigerian clinical setting, demonstrated that the implementation of the NiDST has high fidelity (adherence) (>90%) for patient recruitment, dietary intake assessment and outpatient consultation, suggesting a high adherence to the implementation protocol of the NiDST. These findings align with previous studies on patient-reported outcomes (PROs) among adult cancer and heart failure population studies from countries like the United States, the United Kingdom and the Netherlands, where similar tools have shown comparable average PRO adherence rates ranging from 60% to 85% [50,51,52,53]. The majority of the patients reported that the tool was clear, complete and easy to use and has clinical value as it helps to assess their food intake and supports their clinicians in providing helpful dietary advice. In addition, clinicians and patients demonstrated high engagement levels, with the majority reporting improvement in patient–clinician engagement and communication in dietary discussions, indicating a high quality of delivery of the NiDST. The short completion time (<6 min) and accepted marginal increase in consultation time (<10 min) suggest that the tool can be seamlessly integrated into clinic workflow without significant disruptions. Kristal et al. (2014) also noted minimal disruption to clinic workflows with their dietary screening tool, highlighting its practicality in busy clinical settings with a recommended completion time of ≤15 min) [54]. Moreover, supplementing the tool with dietary intake guidelines (e.g., food intake guideline pamphlets) can support the clinicians in reducing its impact on consultation times and facilitate its usability and value, as suggested by some of the clinicians.

The NiDST effectively addresses the challenges of time constraints, increased workload, and busy clinics by providing a user-friendly, time-efficient tool for assessing patients’ dietary intake, evidenced by the short patient completion and consultation time. Despite the additional increase in consultation time, clinicians acknowledged that the NiDST streamlined the dietary assessment process, allowing for more focused dietary discussions during clinic visits. This aligns with findings from Vadiveloo et al. (2023), where a dietary screening tool facilitated more efficient and focused dietary assessments, improving the overall consultation quality [32]. Additionally, patients reported feeling more engaged in dietary discussions and expressed a greater understanding of their dietary needs and risks of hypertension. Importantly, the NiDST facilitated personalised dietary advice tailored to their needs. This increased engagement and understanding are crucial for fostering healthy behavioural changes, particularly in managing chronic conditions such as hypertension. Similar results were observed by Ardoin et al. (2022), where patients felt more involved in their dietary care, better understood their dietary requirements and risks and were more motivated to have conversations about diet with their physician [55]. These findings highlight the transformative potential of the NiDST in promoting patient–clinician communication, enhancing patient engagement in dietary discussions and fostering healthy behavioural change for hypertension management.

### 4.1. Patient–Clinician Communication

Patient–clinician communication plays a crucial role in effective healthcare delivery, especially in chronic disease management such as hypertension [56]. When augmented by patient-reported outcome measures, including patient-reported dietary intake (NiDST report), it can significantly enhance the quality of care and promote positive patient outcomes [57]. Our study demonstrated that the NiDST enhanced patient–clinician communication by providing focus-guided dietary discussion based on patients’ dietary intake provided by NiDST, enabling clinicians to tailor dietary recommendations according to individual needs. This finding is consistent with a cross-sectional survey by Fabbri et al. (2020), which involved 2398 participants from 11 counties in southeast Minnesota with incident heart failure and a mean follow-up of 1.3 years. The study reported that patients who experienced good or excellent patient-centred communication had a 30% lower risk of death (HR [95%CI]: 0.70 [0.51, 0.97], *p*-trend = 0.020) compared to those with fair or poor patient-centred communication [58].

In addition, patients noted that the use of the NiDST-guided discussion increased their self-awareness of their food intake and empowered them to take control of their dietary habits and make informed positive dietary changes. Furthermore, clinicians reported a positive effect of the NiDST in the sense that it guided the focus of dietary consultation and increased engagement with patients, leading to improved communication quality. This finding aligns with previous studies that reported that the use of patient-reported outcomes during consultations guides the focus of consultations, supports patients’ active engagement and improves communication and positive outcomes. For instance, Mejdahl et al. (2020) found that the application and deliberate use of patient-reported outcome (PRO) measures for epilepsy can affect patient–clinician interaction, promoting patient involvement in terms of improved communication and increased patient activation [59]. Additionally, a systemic review conducted by Chen et al. (2013) with 27 studies reported that the use of PRO measures improved patient–provider communication and patient satisfaction in primary care settings, the monitoring of treatment response and the detection of unrecognised problems [60]. Similarly, another systematic review conducted by Kotronoulas et al. (2014), involving 26 controlled trials, reported that the routine use of PRO measures improved discussion of patient outcomes during consultations and symptom control, increased supportive care measures, and led to higher patient satisfaction leading to improved adherence and better outcomes [61]. Overall, these findings indicated that using patient-reported outcome measures like the NiDST improved patient engagement and patient–clinician communication and underscored the significant role of effective communication in fostering lifestyle change and improvement of patient outcomes, aligning with the significance of patient-reported outcome measures reported by previous studies.

### 4.2. Barriers to and Facilitators of Implementation

To gain insight into factors associated with the implementation of the NiDST, the primary barriers for clinicians were additional time added to consultations (particularly on busy clinic days) and variability in nutritional knowledge in interpreting dietary data. However, as indicated in previous studies, these barriers were mitigated by the simplified, user-friendly design of the NiDST and the brief training received by the participating clinicians at the pre-implementation stage of the study [32]. These findings were consistent with studies that assess the barriers to adopting tools into clinical practice. For instance, a systematic review by Rodrigues et al. (2024) involving 15 studies also identified time constraints and additional training needs as barriers reported by healthcare professionals for the adoption of digital health-related tools for medication appropriateness [62]. Similarly, another systematic review by Wang et al. (2023) involving 20 studies also reported time constraints and a lack of knowledge and skills as common barriers to implementing clinical practice guidelines’ (CPGs) recommendations in primary care [63].

Conversely, facilitators of implementation included the user-friendly design, relevance, compatibility with routine workflow, ease of integration into clinical routine visits, the succinctness of the NiDST questionnaire, short completion time on the use of the NiDST, and the overall favourable reception from patients and clinicians. These findings align with previous studies on the facilitators of the integration of patient-reported outcomes and suggest that providing additional training and nutritional resources for clinicians, sign-posting clinicians to dietician referrals in difficult cases, and leveraging facilitators are important to enhance the implementation of the NiDST. For instance, a study by Palacholla et al. (2019) reported that the key facilitators of digital health technology adoption for hypertension management by physicians were ease of integration with clinical workflow, improvement in patient outcomes, improved self-management and patient experience, and positive impact on patient–provider communication [64].

### 4.3. Practical Implication and Clinical Relevance

This pilot implementation study of the Nigerian Dietary Screening tool (NiDST) underscores its practical application and clinical relevance by demonstrating its ability to facilitate dietary assessments and enhance patient–clinician communication in nutrition education within Nigerian clinical settings. The NiDST’s design addresses several key challenges faced by healthcare systems in low-resource settings, including time constraints, variability in clinicians’ nutritional knowledge and the need for culturally relevant dietary advice. In comparison to existing tools in other countries, the NiDST offers distinct advantages. For instance, in the United States, tools like MyPlate and programmes such as the Diabetes Prevention Program (DPP) provide structured nutrition education and guidelines but often face challenges with consistent use and addressing diverse populations’ needs [65]. Unlike these tools, the NiDST is specifically tailored to the Nigerian context to optimise cultural relevance and ease of integration into routine clinical workflows. This allows for dietary assessments to be seamlessly incorporated into consultations without significant time disruptions. Similarly, the NHS Eatwell Guide in the United Kingdom emphasises preventive care and routine dietary assessments [66]. However, these tools may be too time-consuming for busy clinical settings. The NiDST’s concise format and user-friendly design overcome this limitation, making it more suitable for high-volume clinics where time efficiency is crucial. Importantly, this efficiency did not compromise the quality of dietary discussions, as evidenced by the high levels of patient and clinician satisfaction reported in the pilot study.

In Australia, the Healthy Eating Advisory Service and the Australian Dietary Guidelines provide comprehensive nutritional resources and training for healthcare providers [67]. While effective, these tools require substantial time and resources for full implementation. The NiDST, by contrast, requires only brief training and offers a simplified approach to dietary assessments, making it more feasible for low-resource settings while still delivering effective nutritional guidance. Therefore, the NiDST demonstrates significant potential in transforming dietary assessment and intervention within the Nigerian healthcare system by enhancing patient–clinician communication, supporting personalised dietary advice, and serving as a model for other low-resource settings aiming to improve their nutritional assessment capabilities.

### 4.4. Strengths and Limitations

This study used a mixed-methods approach, integrating quantitative and qualitative inputs from patients and clinic clinicians to understand facilitators and barriers to the implementation of the NiDST. By considering both patients’ and clinicians’ perceptions, the study offers a comprehensive understanding of the fidelity of implementation of the NiDST in addition to the barriers to and facilitators for implementation into routine outpatient consultations in a clinical setting in Nigeria. In short, this single-centre pilot implementation study is an important step to (i) design and plan for the actual implementation of the NiDST across Nigerian clinical settings; (ii) plan and address the barriers identified in this study; and (iii) contribute to further improving and strengthening the value, relevance and acceptability of the NiDST.

Nonetheless, there were some limitations. Firstly, this study did not evaluate the role of organisations and socio-political determinants in this implementation phase because it is a pilot study. However, the evidence from this study will be used to plan for a more robust future implementation study across Nigerian clinical settings. Secondly, the findings may lack generalizability beyond the Nigerian context, as cultural and healthcare system differences could influence the acceptability and integration of the FFQ in other settings. However, adapting the NiDST could improve the generalisation of the tool in other West African countries. Lastly, the lack of long-term follow-up in the study means that it may not capture the sustained effectiveness or sustainability of integrating the NiDST into clinical practice, highlighting the need for a study of longer duration and wider distribution to provide more insights into the NiDST’s continued utility and impact over time. We aim to address this in the next phase of our nationwide implementation study of the NiDST.

## 5. Conclusions

This pilot implementation study demonstrated that the Nigerian dietary screening tool (NiDST) significantly enhances patient–clinician engagement and communication compared to conventional methods of outpatient consultation, lacking structured patient-reported outcome measures. Both patients and clinicians reported positive experiences with the NiDST, highlighting its facilitators and practical benefits in clinical settings. Moreover, the study provided evidence to support the feasibility and potential for widespread clinical adoption of the NiDST across Nigeria’s healthcare system. Integrating NiDST into routine clinical practice could enhance dietary assessments and interventions, enabling personalised advice and more efficient identification of dietary risks crucial for managing conditions like hypertension, and offer a model for low-resource settings to enhance nutritional assessment capabilities and prevention of hypertension and cardiovascular health. These findings lay a solid foundation for further research aimed at optimising the NiDST and expanding its generalizability for implementation in more regions in Nigeria. The NiDST shows great promise in transforming dietary assessment and intervention in Nigerian healthcare, ultimately improving patient health and wellbeing.

## Figures and Tables

**Table 1:** Inclusion and exclusion criteria for patients.

Inclusion Criteria	Exclusion Criteria
Age between 18 and 70 years	Individuals <18 years or >70 years of age
Men and women	Pregnant or breastfeeding women, or those intending to become pregnant
Hypertensive or non-hypertensive individuals	Diagnosis of other chronic diseases such as cancer, diabetes, renal failure, endocrine diseases, and previous and recent incidence of cardiovascular disease (CVD) and stroke
Individuals who have been residents in Nigeria for the past 2 years	Individuals who have been residents in Nigeria for less than 2 years
Ability to read, write, and communicate over the phone in English	Individuals with dietary restrictions or with recent changes to their diet or food
Individuals who gave their consent to participate	Individuals who did not give their consent to participate or were currently enrolled in other studies

**Table 2:** Sociodemographic and clinical characteristics of patients and clinicians.

Characteristics	Overall (*n* = 151)	Hypertensive (*n* = 88, 58.3%)	Non-Hypertensive (*n* = 63, 41.7%)	*p*-Value
**Patients (*n* = 151)**				
Sex, *n* (%)				**0.010**
Male	75 (49.7)	52 (59.1%)	23 (36.5%)	
Female	76 (50.3)	36 (40.9%)	40 (63.5%)	
Age (years)	44.4 ± 11.1	46.0 ± 10.2	42.1 ± 12.2	0.098
Education, *n* (%)				0.472
No formal	1 (0.6)	1 (1.1)	0 (0)	
Primary	26 (17.2)	14 (15.9)	12 (19.1)	
Secondary	62 (41.1)	40 (45.5)	22 (34.9)	
Tertiary	62(41.1)	33 (37.5)	29 (46.0)	
Marital Status, *n* (%)				0.072
Divorced	3 (2.0)	2 (2.3)	1 (1.6)	
Married	119 (78.8)	75 (85.2)	44 (69.8)	
Single	18 (11.9)	6 (6.8)	12 (19.0)	
Widowed	11 (7.3)	5 (5.7)	6 (9.5)	
Employment, *n* (%)				0.057
Employed	25 (16.6)	14 (15.9)	11 (17.5)	
Homemaker	1 (0.6)	0 (0)	1 (1.6)	
Retired	9 (6.0)	7 (8.0)	2 (3.2)	
Self-employed	103 (68.2)	63 (71.6)	40 (63.5)	
Student	8 (5.3)	1(0.6)	7 (11.1)	
Unemployed	5 (3.3)	3 (3.4)	2 (3.2)	
Family history of HTN, *n* (%)	34 (22.5)	26 (29.5)	8 (12.7)	
Physical activity level, *n* (%)				0.542
Active	23 (15.2)	12 (13.6)	11 (17.5)	
Moderately active	15 (9.9)	7 (8.0)	8 (12.7)	
Moderately inactive	41 (27.2)	27 (30.7)	14 (22.2)	
Inactive	72 (47.7)	42 (47.7)	30 (47.6)	
BMI (kg/m^2^)	29.0 ± 6.2	29.1 ± 6.3	29.1 ± 5.7	0.934
Waist circumference (cm)	95.4 ± 14.5	95.49 ± 14.6	96.0 ± 14.2	0.740
Blood pressure (mmHg)				
Systolic blood pressure	142.5 ± 24.2	159.4 ± 15.7	119.0 ± 10.0	**<0.001**
Diastolic blood pressure	98.0 ± 71.5	113.0 ± 9.8	77.1 ± 7.2	**<0.001**
Mean arterial pressur	127.7 ± 32.3	143.9 ± 33.3	105.0 ± 8.0	**<0.001**
NiDST completion time (minutes)	5.5 ± 1.7	5.5 ± 1.6	5.5 ± 1.7	0.905
NiDST consultation time (minutes)	9.6 ± 1.5	9.6 ± 1.5	9.6 ± 1.5	0.920
**Clinicians (*n* = 20)**				
	Overall	Men (*n* = 6)	Women (*n* = 14)	*p*-value
Age (years)	42.6 ± 6.0	43.2 ± 6.0	42.3 ± 6.2	0.771
Years of experience	13.0 ± 4.7	11.2 ± 2.0	13.7 ± 5.4	0.279
Specialty				**<0.001**
Internal Medicine *n* (%)	9 (45)	5 (25)	4 (20)	
Family Medicine *n* (%)	11 (55)	1 (5))	10 (50)	

HTN: hypertension; NiDST: Nigerian diet screening tool.

**Table 3:** Daily intake of 23 food groups assessed among hypertensive and non-hypertensive adults (*n* = 151).

Food Items/Groups (Servings/Day)	Hypertensive (*n* = 88, 58%)			Non-Hypertensive (*n* = 63, 42%)			*p*-Value
	Mean	Median	IQR	Mean	Median	IQR	
**Healthy (non-atherogenic) food groups**							
Fruits	0.36	0.21	0.36	0.39	0.21	0.36	0.687
Vegetables	0.49	0.57	0.36	0.60	0.57	0.79	0.052
Rice and pasta	0.50	0.57	0.36	0.57	0.57	0.58	0.199
Wheat products	0.53	0.57	0.36	0.55	0.57	0.79	0.913
**Fibre-rich cereals**	**0.02**	**0.00**	**0.00**	**0.08**	**0.00**	**0.00**	**0.005**
Beans and lentils	0.38	0.21	0.36	0.44	0.57	0.36	0.199
Nuts and seeds	0.43	0.57	0.36	0.50	0.57	0.36	0.215
White (lean) meat	0.36	0.21	0.36	0.31	0.21	0.36	0.426
Starchy tubers	0.67	0.57	0.43	0.68	0.57	0.43	0.474
Fish and seafoods	0.60	0.57	0.79	0.79	0.57	0.43	0.155
Tea and coffee	0.43	0.57	0.68	0.35	0.21	0.57	0.167
Dairy (Milk)	0.54	0.57	0.79	0.66	0.57	0.79	0.253
**Unhealthy (atherogenic) food groups**							
**Red meat**	**0.66**	**0.57**	**0.43**	**0.43**	**0.57**	**0.36**	**0.001**
**Processed meat**	**0.27**	**0.21**	**0.57**	**0.14**	**0.00**	**0.21**	**0.003**
**Eggs and egg products**	**0.40**	**0.21**	**0.36**	**0.23**	**0.21**	**0.00**	**0.001**
**Fried foods**	**0.49**	**0.57**	**0.36**	**0.29**	**0.21**	**0.36**	**<0.001**
**Fast foods**	**0.40**	**0.21**	**0.57**	**0.26**	**0.00**	**0.57**	**0.005**
**Soups and stews**	**0.83**	**1.00**	**0.43**	**0.68**	**0.57**	**0.43**	**0.027**
**Fats and oils**	**0.94**	**1.00**	**0.43**	**0.77**	**1.00**	**0.43**	**0.035**
**Desserts and sweets**	**0.32**	**0.21**	**0.57**	**0.14**	**0.21**	**0.21**	**0.001**
**Soft drinks**	**0.54**	**0.57**	**0.79**	**0.28**	**0.21**	**0.36**	**<0.001**
**Alcoholic drinks**	**0.51**	**0.21**	**1.00**	**0.12**	**0.00**	**0.21**	**<0.001**
**Salt and seasonings**	**0.92**	**1.00**	**0.43**	**0.71**	**0.57**	**0.43**	**<0.001**

IQR: inter-quartile range.

**Table 4:** Determinants for the use of the NiDST by clinicians (*n* = 20).

MIDI Scale and Determinants	Disagree/ Totally Disagree (%)	Neutral (%)	Agree/ Totally Agree (%)
**Nigerian Dietary Screening Tool**			
**Procedural clarity:** Activities for the use of the NiDST were clearly described	0	0	100
**Completeness:** The food list in the NiDST is comprehensive	0	0	100
**Complexity:** The NiDST is too complex for me to use	100	0	0
**Compatibility:** Compatible with workflows	15	0	85
**Relevance for patient:** Intervention is relevant for my patients	0	0	100
**Clinicians**			
**Personal benefits:** I think using the NiDST will help me to assess patient dietary intake	0	10	90
**Personal communication:** The NiDST will help me to engage and improve engagement and communication with patients	0	0	100
**Outcomes expectations:** I think using the NiDST will support me to offer dietary advice to patients	0	5	95
**Professional obligation:** I feel it is my responsibility to use the NiDST to assess dietary intake and offer dietary advice to patients routinely	0	5	95
**Patient satisfaction:** Patients will be satisfied when using the tool with their dietary intake	0	0	100
**Patient cooperation:** Patient will generally cooperate when using the tool having their dietary intake	0	0	100
**Nutritional knowledge:** I have enough knowledge to use and interpret the dietary assessment tool as intended	25	0	75
**Time available:** I have enough time to assess patient dietary intake and offer dietary counselling	25	0	75

MIDI: Measurement Instrument for Determinants of Innovations.

**Table 5:** Determinants for the use of the NiDST by patients (*n* = 151).

MIDI Scale and Items	Disagree/ Totally Disagree (%)	Neutral (%)	Agree/ Totally Agree (%)
**Personal benefits:** The tool was quick to complete within one clinical visit.	2.0	2.0	96.0
**Personal communication:** Helped me in communicating with doctor.	0.0	0.7	99.3
**Patient engagement:** NiDST helped me discuss with doctors.	0.0	1.3	98.7
**Outcome expectations:** Helped me understand and reflect on my food intake.	0.7	8.6	90.7
**Awareness of content:** The frequency response options allowed me to accurately report my usual food intake.	0.0	4.0	96.0
**Time available:** The time taken to complete the NiDST was acceptable to me.	0.0	0.7	99.3
**Patient satisfaction:** I am satisfied with using the tool and consultation.	1.3	2.7	96.0

MIDI: Measurement Instrument for Determinants of Innovations.

## Data Availability

The dataset presented in this article are not readily available because the data are part of an ongoing study. Requests to access the datasets should be directed to the corresponding author (M.A.Z.).

## Supplementary Materials

The following supporting information can be downloaded at: https://www.mdpi.com/article/10.3390/nu16142286/s1, Table S1: Nigerian Dietary Screening Tool (NiDST) for hypertension; Table S2: Modified version of Measurement Instrument for Determinants of Innovations (MIDI).
