# Supplementary material for: The Nigerian Dietary Screening Tool: A Step toward Improved Patient-Clinician Communication in Nigerian Hospitals: A Pilot Implementation Study
Source: Nutrients. 2024 Jul 16;16(14):2286. doi: 10.3390/nu16142286 (PMC11280457; doi:10.3390/nu16142286)
Supplement: Supplementary file 1 [file nutrients-16-02286-s001.zip › nutrients-3050839-supplementary.pdf]

Supplementary material

The Nigerian dietary screening tool: A step toward improved patient-clinician communication in Nigerian hospitals: A pilot implementation study

Nimisoere P. Batubo[1], Carolyn I. Auma[1], J. Bernadette Moore[1], and Michael A. Zulyniak[1,2,3*]

[1] Nutritional Epidemiology Group, School of Food Science and Nutrition, University of Leeds, Leeds, UK.

[2] Leeds Institute of Medical Research, Faculty of Medicine and Health, University of Leeds, Leeds, UK.

[3] Food, Nutrition and Health, University of British Columbia, Vancouver, BC, Canada.

[*]Corresponding author:

michael.zulyniak@ubc.ca (MAZ)

Author Contributions:

NPB and MAZ collaborated on the research methodology design.

NPB led the trial and the analysis and prepared the first draft.

MAZ provided analytical expertise. MAZ, JBM, and CIA provided critical feedback.

NPB revised the manuscript.

All authors approved the final manuscript.

Table S1: Nigerian Dietary Screening Tool (NiDST) for hypertension

How often do you eat or drink the following foods & drinks in a 'typical' week over the past month or so?
Do not be concerned if some food/snacks or drinks you eat or drink are not mentioned.

Dietary intake information								
S/N	Food group/item	Frequency						
		Never or Rarely	1-2 times/ week	3-5 times/ week	Daily	1-2 times/ day	3-4 times/ day	5+ Times/ day
1	**Fruit** *(such as Mango, Oranges, Pineapples, Pawpaw, Bananas, Guavas, Avocado (peas), Grapefruit, Tangerines, Soursop, Butter pear, watermelon, Cherry, and others)?*	☐	☐	☐	☐	☐	☐	☐
2	**Vegetables** *(such as Cabbage, Carrots, Cucumber, Garden eggs, Eggplant, Broccoli, Corn, Cooked vegetables (e.g. Tomatoes, onions, peppers, bitter leaf, okazi, spinach, pumpkin, ogu), Canned vegetables, and others)?*	☐	☐	☐	☐	☐	☐	☐
3	**Rice, Millet, and Pasta** *(such as Rice, Maise, Millet, Sorghum, Spaghetti, Macaroni, Instant noodles, and others)?*	☐	☐	☐	☐	☐	☐	☐
4	**Wheat products** *(such as Bread, Oat, Semolina,Fonio, Wheat flour)?*	☐	☐	☐	☐	☐	☐	☐
5	**Fibre-rich breakfast cereals** *(such as Cornflakes, GoldenMorn, Weetabix, Fruit 'n Fiber, and others)?*	☐	☐	☐	☐	☐	☐	☐
6	**Beans, and lentils** *(such as Black-eyed peas, Brown beans, Cowpeas, Boiled beans, Moin-moin, and others)?*	☐	☐	☐	☐	☐	☐	☐
7	**Nuts and Seeds** (*such as Groundnut, Coconut, Melon seeds, Sesame seeds, cashews, and others)?*	☐	☐	☐	☐	☐	☐	☐
8	**Red Meat** *(such as Beef, Sheep, Lamb, Pork, Bush meat and others)?*	☐	☐	☐	☐	☐	☐	☐
9	**White Meat** *(such as Chicken, Turkey, Cow head, Cowskin (Kpomo), and others)?*	☐	☐	☐	☐	☐	☐	☐
10	**Processed Meats** *(such as Suya, Kilishi, Sausages, Nkwobi, Bacon, Corned beef, Meat pies, Gala, and others)?*	☐	☐	☐	☐	☐	☐	☐
11	**Eggs and egg products** *(such as Boiled eggs, Fried eggs, Scrambled eggs, and others)?*	☐	☐	☐	☐	☐	☐	☐
12	**Fish and seafood** *(such as Tilapia, Catfish, Mackerel, Shrimp, Stockfish, Smoked fish, and others)?*	☐	☐	☐	☐	☐	☐	☐
13	**Fried foods** *(such as Akara, Puff-puff, Pancakes, Chin-chin, Buns, scotch egg, Chips (fried plantain, yam, and potatoes), Samosa, Fried fish, Fried chicken, Fried beef, and others)?*	☐	☐	☐	☐	☐	☐	☐
14	**Fast (takeaway) foods?** *(e.g., Restaurants foods, local roadside food vendors (Mama put), Shawarma, Pizza, Burgers, fried/jollof rice, fried chicken, Suya, Small chops, Hot dog, Beef/chicken/fish rolls, Doughnuts, and others)*	☐	☐	☐	☐	☐	☐	☐
15	**Starchy tubers** *(such as Yam, Cassava, Plantain, Cocoyam, Potatoes, and others)?*	☐	☐	☐	☐	☐	☐	☐
16	**Soups and Stew (sauce)** *(such as Egusi, Banga, or groundnut, vegetable, Afang, Edikaikong, Bitter leaf, Efo-Riro, Okra, Ogbono, Ewedu, Fisherman, white, Nsala soups and others), and (palm oil stew, vegetable oil stew, sauces, and others)?*	☐	☐	☐	☐	☐	☐	☐
17	**Fats and Oils** *(such as food cooked with palm oil, vegetable oil, butter/margarine/ mayonnaise, Lard, shea butter, salad cream, and others)?*	☐	☐	☐	☐	☐	☐	☐
18	**Dessert and sweets** *(Ice cream, yoghurt, cheese, Biscuits, cakes, chocolate, sweets, and others?)*	☐	☐	☐	☐	☐	☐	☐
19	**Fizzy (Soft) drinks and fruit juices** *(such as Coke, Fanta, Sprite, Pepsi, Malt, Maltina, Zobo, Tiger nut, Kunu, smoothies, 5live, Pineapple juice, energy drinks and others)?*	☐	☐	☐	☐	☐	☐	☐
20	**Alcoholic drinks** *(such as Beer, wine, gin, whisky, spirit, liquor, champagne, and others)?*	☐	☐	☐	☐	☐	☐	☐
21	**Tea and Coffee**	☐	☐	☐	☐	☐	☐	☐

	(such as green tea, black tea, Herbal tea, Lipton, Nescafe, and others)?							
**22**	**Dairy- Milk or milk-based beverages**<br>*(such as Peak, Three Crowns, Dano, Cowbell, Loya Milk, Coast, Fura da nonon, Hollandia, Nunu, Kunun zaki, Milo, Ovaltine, Chocomilo, Nesquik, Nutri-C, Viju Milk, smoothies, and others)?*	☐	☐	☐	☐	☐	☐	☐
**23**	**Seasonings**<br>*(such as Salt, Seasoning cubes (e.g., Maggi, Knorr), Sauce (e.g., Soy sauce, Suya sauce, Fish sauce, Oyster sauce), and others)*	☐	☐	☐	☐	☐	☐	☐
**24**	**Salt use**<br>*How often do you add extra salt to your food at the table when eating?*	**Never or rarely**<br>☐		**Sometimes**<br>☐				**Always**<br>☐

**25**	**What type of milk do you usually drinks?**	Skim milk ☐	Low-fat milk ☐	Whole milk ☐

Table S2: Modified version of Measurement Instrument for Determinants of Innovations (MIDI)

This section will ask you to provide any comments, concerns, or suggestions you may have regarding the questionnaire. Your feedback is valuable to us and will help improve the quality of the dietary assessment tool.

You can fill out the questionnaire by placing a "tick" in the box next to your answer based on the response scale.

S/N	Determinants	Response scale				
		Totally disagree	Disagree	Neutral	Agree	Totally agree
**Section 1: Determinant associated with the innovation-Nigerian dietary screening tool (NiDST)**						
1	**Procedural clarity:** The NiDST clearly describes the activities I should perform and in which order					
2	**Completeness:** The food list in the NiDST is comprehensive					
3	**Complexity:** The NiDST is too complex for me to use. The questions were not clear or easy to understand					
4	**Compatibility:** The NiDST is a good match for how I used to work (i.e., compatible with my workflows)					
5	**Relevance for patient:** I think the NiDST is relevant for my patients					
**Section 2: Determinants associated with the clinicians**						
1	**Personal benefits:** I think the NiDST supported me to assess patient dietary intake					
2	**Personal communication:** I think using the NiDST improved my engagement and communication with patients					
3	**Outcome expectation:** I think using the patient-reported NiDST supported me to offer dietary advice to patients					
4	**Professional obligation:** I feel it is my responsibility to use the NiDST to assess dietary intake and offer dietary advice to patients routinely					
5	**Patient satisfaction:** Patients will generally be satisfied if I use their completed NiDST for their dietary discussion					
6	**Patient cooperation:** Patient will generally cooperate if I use their completed NiDST in dietary discussion					
7	**Nutritional knowledge:** I have enough knowledge to use and interpret the NiDST as intended					
8	**Time available:** I have enough time to use the completed NiDST for dietary discussion in outpatient clinic					
**Section 3: Determinants associated with the patients**						
1	**Complexity:** The NiDST is too complex for me to complete. The questions were clear or easy to understand					
2	**Personal benefit:** I think the NiDST help me to understand and reflect on my food intake					
3	**Patient engagement:** I think the NiDST help me discuss with doctors about my food					
4	**Outcome expectations:** I think the NiDST Help me discuss with doctors					
5	**Awareness of content:** I think food list and frequency response options allow me to accurately report my usual food intake					
6	**Time available:** The time taken to complete the NiDST was acceptable to me during waiting for my consultation					
7	**Patient satisfaction:** I am satisfied with using the NiDST during outpatient consultation					
8	**Personal communication:** I think using the NiDST improved my communication with doctor during outpatient consultation					

Thank you for participating in this study.
